# Dual Primary Gastric and Rectal Adenocarcinoma: A Case Report with Management Insights: Dual Primary Gastric and Rectal Adenocarcinoma

**DOI:** 10.31661/gmj.v14i.3742

**Published:** 2025-07-07

**Authors:** Ali Dastjerdi, Elham Samami, Seyed Alireza Javadinia, Maryam Anvary, Asghar Kazemzadeh, Mehdi Molavi, James S Welsh

**Affiliations:** ^1^ Student Research Committee, Sabzevar University of Medical Sciences, Sabzevar, Iran; ^2^ States and College of Nursing, University of Florida, Gainesville, FL, United States; ^3^ Non-Communicable Diseases Research Center, Sabzevar University of Medical Sciences, Sabzevar, Iran; ^4^ Department of Internal Medicine, Mashhad University of Medical Sciences, Mashhad, Iran; ^5^ Cellular and Molecular Research Center, Sabzevar University of Medical Sciences, Sabzevar, Iran; ^6^ Loyola University Chicago Stritch School of Medicine, Edward Hines Jr., VA Hospital, Department of Radiation Oncology Maywood, IL, USA

**Keywords:** Dual Primary Malignancies, Colorectal Cancer, Gastric Cancer, Metachronous, Synchronous, Multidisciplinary Team

## Abstract

**Background:**

Dual primary malignancies, including colorectal (CRC) and gastric cancers (GC), are complicated cases due to the complexity of managing patients.

**Case Report:**

This case report presents a 62-year-old male patient with rectal and gastric adenocarcinomas. Initially, rectal adenocarcinoma after a complaint of hematochezia was diagnosed by prognostic modalities. The patient received total neoadjuvant therapy with FOLFOX chemotherapy and chemoradiotherapy. After surgery, a complete pathological response was obtained. A few months later, gastric adenocarcinoma with persistent heartburn was detected through esophagogastroduodenoscopy (EGD). total neoadjuvant therapy with FOLFOX chemotherapy and chemoradiotherapy followed by total gastrectomy were prescribed. After gastrectomy, a complete pathological response was obtained.

**Conclusion:**

This case of synchronous CRC and GC, diagnosed 5 months apart, underscores the pivotal role of early detection and multidisciplinary management in achieving favorable outcomes. Complete pathologic responses in both malignancies following tailored TNT with FOLFOX and FLOT regimens, combined with surgical interventions, highlight the efficacy of personalized treatment strategies, even in resource-constrained settings. Continued research is essential to optimize diagnostic protocols, refine therapeutic approaches, and improve access to genetic testing for synchronous and metachronous malignancies, promoting equitable cancer care globally.

## Introduction

Multiple primary malignancy (MPM) is defined as the presence of at least two primary malignancies in a single patient [1]. MPM can be divided into synchronous (detection within six months from the first diagnosis) and metachronous (detection after six months from the first diagnosis) [2]. The incidence rate of MPM among all malignancies is reported to be approximately 0.7% to 11.7% [3][4][5].

CRC and GC are among the most prevalent secondary cancers for each other [6], with the reported incidence rate of GC in patients with CRC ranging from 2% to 2.4% [7][8][9]. Similarly, the most commonly reported second primary cancer among GC patients is colorectal adenocarcinoma [10]. In DPGCC, synchronous cancers have a worse prognosis compared to metachronous cancers [11]. Metachronous cancers exhibit a better treatment response for the first primary cancer. This is perhaps due to the increased surveillance following treatment of the first cancer, which allows the second cancer to be detected at an earlier stage and in time [12]. Alternatively, the explanation might lie in unexplained biological differences between the synchronous and metachronous groups. One study found that patients with metachronous DPGCC tended to be younger, had fewer comorbidities and had significantly higher 5-year overall survival rates than those with synchronous DPGCC [13]. Although the decision to undergo surgery can be difficult, early diagnosis and surgical resection are crucial factors in achieving better outcomes for patients with DPGCC [11]. This case study aims to improve the awareness and management of dual primary cancers, specifically focusing on the prognosis and treatment of CRC and GC.

## Case presentation

**Table:** Table1. Chronological Overview of Diagnostic and Therapeutic Events

Date	Event	Details
July 2023	Rectal cancer diagnosis	Colonoscopy and biopsy confirmed well-differentiated adenocarcinoma (cT2N0M0).
August-October 2023	Neoadjuvant therapy for rectal cancer	Total neoadjuvant therapy (TNT): FOLFOX (8 cycles: oxaliplatin 85 mg/m², leucovorin 200 mg/m², 5-fluorouracil 400 mg/m² bolus and 2400 mg/m² infusion) + chemoradiation (50.4 Gy in 28 fractions with capecitabine 825 mg/m² twice daily).
November 2023	Rectal cancer surgery	Low anterior resection with lateral lymph node dissection and temporary colostomy; pathology showed complete response (ypT0N0M0).
December 2023	Gastric cancer diagnosis	EGD identified well-differentiated adenocarcinoma in the cardia extending to the lesser curvature (cT3N1M0); confirmed by biopsy, EUS, and CT scan.
January-March 2024	Neoadjuvant therapy for gastric cancer	FLOT chemotherapy (4 cycles: docetaxel 50 mg/m², oxaliplatin 85 mg/m², leucovorin 200 mg/m², 5-fluorouracil 2600 mg/m²) + chemoradiation (paclitaxel 50 mg/m², carboplatin AUC 2).
April 2024	Gastric cancer surgery	Total gastrectomy, distal esophagectomy, and D2 lymph node dissection; pathology confirmed complete response (ypT0N0M0).

A 62-year-old male patient with a history of controlled diabetes mellitus was admitted to the hospital due to hematochezia in July 2023, for six months. The blood tests were normal except for iron deficiency anemia. The colonoscopic revealed an ulcerative rectal lesion located two to eight cm above the anal verge, but the procedure was incomplete due to partial obstruction. A metastatic workup showed circumferential enhancement, but no distant metastasis was identified on the intravenous contrast-enhanced whole-body computed tomography (CT) scan. Liver and renal function tests were within their respective normal limits and preoperative serum concentrations of carcinoembryonic antigen (CEA) and carbohydrate antigen 19-9 (CA 19-9) were also normal. Due to the obstructive mass causing rectal stenosis, pelvic MRI with endorectal coil and endorectal ultrasound (ERUS) were not performed. A biopsy of the lesion confirmed a diagnosis of well-differentiated adenocarcinoma. Genetic testing for hereditary and genetic syndromes was not performed due to financial constraints and the patient’s reluctance.

Following initial evaluations, the patient began treatment with a total neoadjuvant therapy (TNT) protocol. This included whole pelvic chemoradiotherapy at 50.4 Gy in 28 fractions (with concurrent capecitabine at 825 mg/m² twice daily) and the FOLFOX regimen over eight cycles [oxaliplatin 85 mg/m² on day 1, leucovorin 200 mg/m² on day 1, 5-fluorouracil 400 mg/m² on day 1, and 5-fluorouracil 2400 mg/m² as a 48-hour infusion every two weeks]. One month after completing neoadjuvant therapy, the patient underwent low anterior resection and lateral lymph node dissection with a temporary colostomy. The pathology report from the surgery specimen indicated complete pathologic responses in both the primary tumor and regional lymph nodes (ypT0N0M0). After the initial diagnosis of rectal adenocarcinoma, when the patient was readmitted for closure of the temporary loop colostomy in December 2023, he reported persistent heartburn unresponsive to H2 blockers and proton pump inhibitors (PPIs). An esophagogastroduodenoscopy (EGD) revealed an ulcerative malignant lesion in the cardia extending to the lesser curvature of the stomach. The biopsy of the above-mentioned lesion confirmed the diagnosis of well-differentiated adenocarcinoma in the cardia and proximal stomach. Further molecular and immunohistochemistry assessments showed no PD-L1 and HER2 expression and stable mismatch repair (MMR).

Subsequent endoscopic ultrasound (EUS) and a contrast-enhanced CT scan of the abdomen and pelvis were performed. The EUS report showed diffuse mucosal thickening of the gastric cardia and lower esophageal sphincter (LES) containing a malignant lesion with a thickness of 11 mm and involvement of two regional lymph nodes. The CT scan report revealed no remarkable data or evidence of distant metastasis. The staging of gastric cancer was cT3N1M0.

A multidisciplinary team (MDT) recommended neoadjuvant chemotherapy and chemoradiotherapy for gastric cancer. The patient was prescribed FLOT chemotherapy [docetaxel 50 mg/m2, oxaliplatin 85 mg/m2, leucovorin 200 mg/m2, and fluorouracil 2600 mg/m2 as a 24-h infusion on day 1] followed by chemoradiotherapy(with concurrent paclitaxel at the dose of 50 mg/m2 and carboplatin with AUC of 2) was prescribed.

Three months later, the patient underwent total gastrectomy, distal esophagectomy, and D2 lymph node dissection. Pathologic evaluation showed a pathologic complete response in the primary tumor and regional lymph nodes (ypT0N0M0).

Table-1 provides a chronological summary of the key diagnostic and therapeutic events. This timeline outlines the sequence of interventions, from the initial diagnosis of rectal adenocarcinoma in July 2023 to the complete pathological responses achieved for both malignancies by April 2024. It highlights the multidisciplinary approach, including neoadjuvant therapies (FOLFOX and FLOT regimens) and surgical interventions (low anterior resection and total gastrectomy), which were critical to the successful management of this complex case.

## Ethical Considerations

This case report was approved by the Institutional Ethics Committee of Sabzevar University of Medical Sciences, dated January 7, 2025, under approval code IR.MEDSAB.REC.1403.145. Written informed consent was obtained from the patient for the publication of this case report and any accompanying data, in accordance with the Declaration of Helsinki. All patient information was anonymized to ensure confidentiality.

## Discussion

Managing synchronous and metachronous dual primary cancers, such as CRC and GC, presents significant challenges in clinical practice. In this case, the synchronous diagnosis of rectal adenocarcinoma followed by gastric adenocarcinoma 5 months later highlights the critical need for vigilant surveillance in patients with gastrointestinal malignancies. The initial rectal cancer, identified via colonoscopy prompted by hematochezia, was effectively managed with TNT using FOLFOX chemotherapy and chemoradiotherapy, followed by low anterior resection, achieving a complete pathologic response. The subsequent detection of GC, triggered by persistent heartburn and confirmed through EGD, underscores the importance of symptom-driven evaluations in CRC patients to identify synchronous malignancies early.

Early detection of synchronous cancers significantly improves patient outcomes [13][14]. EGD, should be considered in patients diagnosed with CRC due to the documented increased risk of a parallel malignancy within the GI tract. Studies report that synchronous CRC occurs in 1.1% to 8.1% of colorectal cancer patients, with specific studies estimating rates of approximately 5.6% to 5.7% depending on diagnostic methods used [15][16]. GC is the most commonly associated second primary cancer in patients with CRC, with an incidence of 2% to 2.4% reported in this population [8][9]. A prospective study in Korea found that 2% of CRC patients had synchronous gastric cancer, with 83.9% detected at an early stage via preoperative EGD, enabling minimally invasive treatments like endoscopic mucosal resection in many cases [8]. These findings strongly support incorporating routine EGD into the diagnostic workup of CRC patients, particularly when gastrointestinal symptoms arise, to facilitate early detection of synchronous gastric cancers.

In this study, due to previously noted relative rectal stenosis, MRI was not feasible, and although PET or PET/CT could have been impactful, its use was precluded by guideline non-recommendation and limited instrumental resources [17][18]. However, it should be noted that PET has variable sensitivity in detecting GC, particularly in early-stage cases due to low detection rates and variable uptake influenced by the histological subtype [19]. In addition, PET/CT imaging provides an enhanced positive predictive value for lymph node metastasis and demonstrates superior sensitivity compared to CT alone [19]. The amalgamation of PET and CT imaging proves particularly advantageous for the assessment of distant metastases [19]. The differentiation between metastatic disease and a second primary cancer holds significant importance, as it directly impacts the therapeutic strategy and overall prognosis. In this framework, the MDT decided to adopt a practical and accessible resource, that is, the contrast-enhanced CT scan, to aid their diagnostic evaluations.

Hereditary cancer syndromes, such as Lynch syndrome, familial adenomatous polyposis, Peutz-Jeghers syndrome, and hereditary gastric cancer, driven by mutations in APC, MMR, STK11, or CDH1 genes, increase the risk of dual primary cancers [20]. Genetic testing is essential for risk stratification and guiding surveillance for patients and their families, yet financial barriers and patient reluctance, as seen in this case, often hinder its adoption [21][22]. Regardless of the recent decrease in genetic testing costs and the improvements in insurance support, many patients continue to decline testing, especially when it doesn’t influence their present therapeutic approach promptly. Nevertheless, genetic results can affect treatment recommendations; for instance, individuals with PALB2, CHEK2, or TP53 mutations are generally counseled against radiation therapy owing to associated risks, although investigations in this domain are still evolving [23][24][25]. In the current case, the patient opted against genetic testing due to financial concerns and personal hesitance, and this highlights the persistent challenge of persuading patients to adopt genetic knowledge for comprehensive surveillance and familial risk management.

Therapeutic decisions for synchronous CRC and GC require nuanced judgment. The initial diagnosis of rectal adenocarcinoma in this patient led to the administration of the TNT protocol, which included FOLFOX chemotherapy and chemoradiotherapy, and underwent surgical intervention. However, after the diagnosis of GC, the question arose whether the patient, who had already undergone chemotherapy containing 5-fluorouracil and oxaliplatin, should proceed directly to surgery or, given the passage of time and the potential growth of the tumor, should undergo to chemotherapy with the FLOT regimen and chemoradiotherapy before gastrectomy. Studies have shown greater efficacy of FLOT chemotherapy in treating GC [26]. While both the FOLFOX and FLOT regimens contain fluorouracil, leucovorin, and oxaliplatin, the addition of docetaxel in the FLOT regimen has been associated with improved survival outcomes in patients with gastric cancer [26][27][28]. Based on the MDT’s assessment, switching to the FLOT regimen was deemed appropriate to maximize the therapeutic benefit before surgery. This decision aligns with current evidence indicating that FLOT is preferable to FOLFOX for neoadjuvant treatment of resectable gastric cancer [26][29][30].

The successful pathologic results post-surgery in this patient demonstrate the efficacy of the chosen therapeutic strategy. However, continuous close monitoring and flexibility in adjusting the treatment plan remain essential, particularly when managing synchronous malignancies that may respond differently to the same chemotherapy regimen.

The management of dual primary gastric and rectal adenocarcinomas in this case highlights the critical role of MDT in orchestrating precise diagnostics and tailored treatments, achieving complete pathologic responses for both malignancies. By integrating expertise from surgery, medical oncology, radiotherapy, and nursing, the MDT enabled informed decisions. This collaboration enhanced coordination and minimized errors, with studies indicating that 31% of treatment decisions are refined through MDT discussions [31]. The case outcomes align with data reporting a 3-year disease-free survival of 78% in MDT-managed patients versus 65% in non-MDT groups, alongside a 2-year survival increase from 58.6% to 65% [32][33]. Despite challenges, such as the patient’s refusal of genetic testing due to financial concerns, the MDT maintained a patient-centric approach, demonstrating flexibility in treatment planning, though fully integrating patient preferences remains an area for improvement [34]. This case exemplifies the MDT’s strength in overcoming diagnostic and therapeutic barriers to optimize outcomes in complex metachronous cancer scenarios.

The pathologic outcome, specifically the complete pathologic response in both the primary tumor and regional lymph nodes underscores the effectiveness of the multimodal treatment approach. Achieving a complete pathologic response is a strong predictor of favorable long-term outcomes in patients with gastric cancer. This case illustrates that with appropriate diagnostic and therapeutic interventions, even patients with synchronous cancers can achieve excellent outcomes.

### Limitations

This case report has several limitations that merit consideration. Firstly, genetic testing to evaluate hereditary cancer syndromes, such as Lynch syndrome or familial adenomatous polyposis, was not performed due to financial constraints and the patient’s reluctance. The absence of molecular profiling limits our understanding of potential genetic predispositions, which could have informed long-term surveillance strategies for the patient and risk assessment for family members. Secondly, the inability to conduct pelvic MRI or endorectal ultrasound (ERUS) due to rectal stenosis restricted precise pretreatment staging of the rectal tumor. This may have impacted the granularity of therapeutic planning, although contrast-enhanced CT provided sufficient staging information. Thirdly, advanced imaging modalities, such as PET/CT, were not utilized during the initial diagnostic workup due to resource limitations. PET/CT could have potentially facilitated earlier detection of the gastric malignancy, given its higher sensitivity for lymph node and distant metastases compared to CT alone [19]. Finally, as a single case report, the findings are inherently limited in generalizability, and the short-term follow-up period precludes assessment of long-term outcomes, such as recurrence or overall survival.

To address these limitations within the current study, the multidisciplinary team relied on accessible diagnostic tools, including colonoscopy, esophagogastroduodenoscopy, and contrast-enhanced CT, which proved effective in achieving complete pathological responses for both malignancies. Comprehensive clinical evaluations and tumor board discussions further compensated for the lack of advanced imaging and genetic data, ensuring tailored treatment decisions. For future studies, implementing cost-effective screening protocols, such as routine EGD in patients diagnosed with colorectal cancer, could enhance early detection of synchronous gastrointestinal malignancies. Additionally, advocating for subsidized genetic testing programs or expanded insurance coverage would improve access to critical risk stratification tools, enabling personalized surveillance and preventive strategies. Incorporating PET/CT or other advanced imaging modalities in resource-available settings could further refine diagnostic accuracy. Lastly, prospective cohort studies with extended follow-up periods are essential to evaluate the long-term efficacy of neoadjuvant therapies and surveillance protocols in patients with dual primary cancers. These strategies would mitigate diagnostic and therapeutic barriers, ultimately improving patient outcomes and contributing to broader clinical knowledge.

## Conclusion

This case report of synchronous dual primary CRC and GC, with gastric adenocarcinoma diagnosed 5 months after rectal adenocarcinoma, illustrates the heightened risk of secondary gastrointestinal malignancies in CRC patients. The complete pathologic responses achieved in both cancers following TNT with FOLFOX for CRC and FLOT for GC, alongside low anterior resection and total gastrectomy, demonstrate the success of individualized treatment strategies. These outcomes are consistent with studies emphasizing that early detection through symptom-driven surveillance, such as EGD for persistent heartburn, significantly improves prognosis in synchronous cancers [4][7][11]. Collaborative care, integrating surgical, medical, and radiation oncology expertise, was instrumental in navigating the complexities of sequential diagnoses and treatments in a resource-constrained setting. The absence of genetic testing due to financial constraints highlights a critical barrier to comprehensive risk assessment and familial counseling [19][20]. Clinicians should maintain heightened awareness for secondary malignancies in CRC patients, particularly when new symptoms arise, and consider timely diagnostic evaluations like endoscopy. Future research should focus on developing cost-effective screening strategies, standardizing therapeutic protocols for synchronous cancers, and addressing socioeconomic barriers to genetic testing to enhance early detection, optimize outcomes, and ensure equitable access to care worldwide.

## Conflict of Interest

All authors declare that they have no potential conflicts of interest including financially or nonfinancially, directly or indirectly related to the work.

## References

[R1] Warren S, Gates O (1932). Multiple primary malignant tumors A survey of the literature and
a statistical study. Am J cancer.

[R2] Suzuki T, Takahashi H, Yao K, Inagi K, Nakayama M, Makoshi T (2002). Multiple primary malignancies in the head and neck: a clinical
review of 121 patients. Acta Oto-Laryngologica.

[R3] Artac M, Bozcuk H, Ozdogan M, Demiral AN, Sarper A, Samur M (2005). Different clinical features of primary and secondary tumors in
patients with multiple malignancies. Tumori Journal.

[R4] Demandante CG, Troyer DA, Miles TP (2003). Multiple primary malignant neoplasms: case report and a
comprehensive review of the literature. American journal of clinical oncology.

[R5] Tachimori Y (2002). Cancer screening in patients with cancer. Japanese journal of clinical oncology.

[R6] Ferlay J, EM, Lam F, Laversanne M, Colombet M, Mery L, Piñeros M, Znaor A, Soerjomataram I, Bray F (2024). Lyon, France:International Agency for Research on Cancer,Global Cancer Observatory: Cancer Today (version 1.1).

[R7] Lee JH, Bae JS, Ryu KW, Lee JS, Park SR, Kim CG (2006). Gastric cancer patients at high-risk of having synchronous
cancer. World journal of gastroenterology.

[R8] Lim SB, Jeong SY, Choi HS, Sohn DK, Hong CW, Jung KH (2008). Synchronous gastric cancer in primary sporadic colorectal cancer
patients in Korea. International journal of colorectal disease.

[R9] Yoon SN, Oh ST, Lim SB, Kim TW, Kim JH, Yu CS (2010). Clinicopathologic characteristics of colorectal cancer patients
with synchronous and metachronous gastric cancer. World journal of surgery.

[R10] Saito S, Hosoya Y, Togashi K, Kurashina K, Haruta H, Hyodo M (2008). Prevalence of synchronous colorectal neoplasms detected by
colonoscopy in patients with gastric cancer. Surgery today.

[R11] Lin Y-J, Chen H-X, Zhang F-X, Hu X-S, Huang H-J, Lu J-H (2023). Features of synchronous and metachronous dual primary gastric and
colorectal cancer. World Journal of Gastrointestinal Oncology.

[R12] Park J-H, Baek J-H, Yang J-Y, Lee W-S, Lee W-K (2018). Clinicopathologic characteristics and survival rate in patients
with synchronous or metachronous double primary colorectal and gastric
cancer. Korean Journal of Clinical Oncology.

[R13] Engstrand J, Strömberg C, Nilsson H, Freedman J, Jonas E (2019). Synchronous and metachronous liver metastases in patients with
colorectal cancer—towards a clinically relevant definition. World Journal of Surgical Oncology.

[R14] Tanjak P, Suktitipat B, Vorasan N, Juengwiwattanakitti P, Thiengtrong B, Songjang C (2021). Risks and cancer associations of metachronous and synchronous
multiple primary cancers: a 25-year retrospective study. BMC Cancer.

[R15] Flor N, Zanchetta E, Di Leo, Mezzanzanica M, Greco M, Carrafiello G, et al (2018). Synchronous colorectal cancer using CT colonography vs other
means: a systematic review and meta-analysis. Abdominal radiology (New York).

[R16] He W, Zheng C, Wang Y, Dan J, Zhu M, Wei M (2019). Prognosis of synchronous colorectal carcinoma compared to
solitary colorectal carcinoma: a matched pair analysis. European journal of gastroenterology & hepatology.

[R17] Benson AB, Venook AP, Adam M, Chang G, Chen Y-J, Ciombor KK (2024). NCCN Guidelines® Insights: Rectal Cancer, Version 3.2024:
Featured Updates to the NCCN Guidelines. Journal of the National Comprehensive Cancer Network.

[R18] Cervantes A, Adam R, Roselló S, Arnold D, Normanno N, Taïeb J (2023). Metastatic colorectal cancer: ESMO Clinical Practice Guideline
for diagnosis, treatment and follow-up. Annals of Oncology.

[R19] Atay-Rosenthal S, Wahl RL, Fishman EK (2012). PET/CT findings in gastric cancer: potential advantages and
current limitations. Imaging in Medicine.

[R20] Shenoy S (2024). Synchronous gastric and colon cancers: Important to consider
hereditary syndromes and chronic inflammatory disease associations. World J Gastrointest Oncol.

[R21] Ajose AO, Graef K, Mallum AA, Oladipo A, Ibrahim F, Li H (2024). Genetic testing in cancer care: An assessment of current practice
in Africa. Journal of Clinical Oncology.

[R22] The Lancet (2023). Meeting the need for germline testing. The Lancet Oncology.

[R23] Ho V, Chung L, Lim SH, Ma Y, Wang B, Lea V (2022). Prognostic Impact of TP53 Mutations and Tumor Mutational Load in
Colorectal Cancer. Gastrointestinal Disorders.

[R24] Infante M, Arranz-Ledo M, Lastra E, Olaverri A, Ferreira R, Orozco M (2024). Profiling of the genetic features of patients with breast,
ovarian, colorectal and extracolonic cancers: Association to CHEK2 and PALB2
germline mutations. Clinica chimica acta; international journal of clinical chemistry.

[R25] Li Y, Liu H, Li T, Feng J, He Y, Chen L (2021). Choroid Plexus Carcinomas With TP53 Germline Mutations:
Management and Outcome. Frontiers in oncology.

[R26] Farrokhi P, Sadeghi A, Sharifi M, Riechelmann R, Moghaddas A (2022). Efficacy and safety of FLOT regimen vs DCF, FOLFOX, and ECF
regimens as perioperative chemotherapy treatments for resectable gastric
cancer patients; a report from the middle east. Research in pharmaceutical sciences.

[R27] Möhring C, Timotheou A, Mańczak A, Sadeghlar F, Zhou T, Mahn R (2023). Efficacy and tolerability of fluorouracil, leucovorin,
oxaliplatin and docetaxel (FLOT) in unselected patients with advanced
gastric and gastroesophageal cancer: does age really matter. Journal of cancer research and clinical oncology.

[R28] Tastekin D, Paksoy N, Dogan I, Ferhatoglu F, Khanmammadov N, Bozbey HU, et al (2023). Fluorouracil, leucovorin, oxaliplatin, and docetaxel (FLOT)
regimen in the first-line treatment of metastatic gastric cancer: A
single-center experience. J Cancer Res Ther.

[R29] Dos Santos, Lequesne J, Leconte A, Corbinais S, Parzy A, Guilloit J-M (2022). Perioperative treatment in resectable gastric cancer with
spartalizumab in combination with fluorouracil, leucovorin, oxaliplatin and
docetaxel (FLOT): a phase II study (GASPAR). BMC Cancer.

[R30] Sah BK, Zhang B, Zhang H, Li J, Yuan F, Ma T (2020). Neoadjuvant FLOT versus SOX phase II randomized clinical trial
for patients with locally advanced gastric cancer. Nature Communications.

[R31] Anghelone A, Bensi M, Barbaro B, Calegari MA, Cina C, Menghi R, et al The impact of the multidisciplinary team (MDT) in the management of colorectal cancer (CRC).

[R32] Layfield DM, Flashman KG, Benitez Majano S, Senapati A, Ball C, Conti JA, et al (2022). Changing patterns of multidisciplinary team treatment, early mortality, and survival in colorectal cancer. BJS open.

[R33] Mangone L, Zizzo M, Nardecchia M, Marinelli F, Bisceglia I, Braghiroli MB (2024). Impact of Multidisciplinary Team Management on Survival and
Recurrence in Stage I–III Colorectal Cancer: A Population-Based Study in
Northern Italy. Biology.

[R34] Ostroff C (2024). Multidisciplinary teams and social science: a patient
perspective. Colorectal Disease.

